# Evaluation of population-specific polygenic risk scores for blood lipids: insights from Taiwanese cohorts and multiancestry meta-analysis

**DOI:** 10.3389/fbinf.2026.1823599

**Published:** 2026-08-24

**Authors:** Yu-Chia Chen, Ting-Yuan Liu, Chi-Chou Liao, Jai-Sing Yang, Wei-De Lin, Wen-Ling Liao, Shih-Yin Chen, Fuu-Jen Tsai

**Affiliations:** 1 Million-Person Precision Medicine Initiative, Department of Medical Research, China Medical University Hospital, Taichung, Taiwan; 2 Master Program for Digital Health Innovation, China Medical University, Taichung, Taiwan; 3 Department of Medical Research, China Medical University Hospital, Taichung, Taiwan; 4 School of Post Baccalaureate Chinese Medicine, China Medical University, Taichung, Taiwan; 5 Center for Personalized Medicine, China Medical University Hospital, Taichung, Taiwan; 6 Graduate Institute of Integrated Medicine, College of Chinese Medicine, China Medical University, Taichung, Taiwan; 7 School of Chinese Medicine, College of Chinese Medicine, China Medical University, Taichung, Taiwan; 8 Departments of Medical Genetics, China Medical University Hospital, Taichung, Taiwan

**Keywords:** blood lipid profile, gene polymorphism, genome-wide association study, LDL-C/TC ratio, multiancestry meta-analysis

## Abstract

**Background:**

Blood lipids are heritable risk factors for cardiovascular disease (CVD), a leading cause of mortality worldwide. However, the genetic architecture of lipid traits and the performance of polygenic risk scores (PRSs) remain underexplored in East Asian (EAS) populations, including Taiwanese Han individuals.

**Methods:**

We conducted genome-wide association studies and PRS analyses for five lipid traits: total cholesterol, high-density lipoprotein cholesterol, low-density lipoprotein cholesterol, triglycerides, and the ratio of low-density lipoprotein cholesterol to total cholesterol. Lipid profile data were obtained from the China Medical University Hospital cohort. PRSs were evaluated on the basis of their correlations with measured lipid levels. To evaluate trans-ancestry PRS transferability, localized models were systematically compared against models derived from discovery-stage GWAS meta-analyses incorporating five ancestry groups from the Global Lipids Genetics Consortium. The performance of the PRS models in predicting lipid-related diseases was evaluated through receiver operating characteristic curve analyses.

**Results:**

The population-specific PRS models explained 11%–40% of the variance in lipid levels within the target cohort. Models leveraging global multiancestry GWAS meta-analysis weights revealed limited predictive performance (*r* ^2^ = 0.04–0.19), whereas analyses incorporating EAS-specific data yielded higher correlations (*r* ^2^ = 0.13–0.30), although these correlations did not exceed those derived from the hospital-based cohort alone. When combined with age and sex, the PRS models demonstrated strong predictive performance for coronary artery disease, atherosclerosis, and ischemic stroke, with area under the curve values of 0.910, 0.926, and 0.854, respectively.

**Conclusion:**

Population-specific PRS models derived from a Taiwanese population outperformed meta-analysis-derived frameworks in predicting lipid levels and demonstrated substantial potential for predicting CVD risk, indicating the importance of ancestry-matched genetic studies in precision medicine.

## Graphical Abstract

**Figure:**
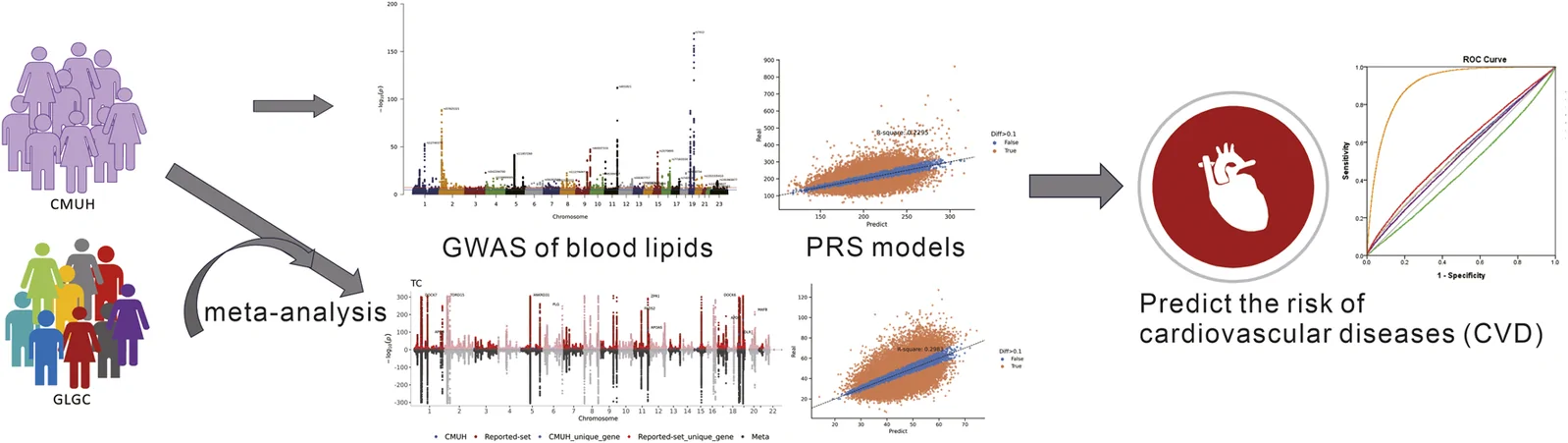

## 1 Introduction

Blood lipids are heritable risk factors for cardiovascular disease (CVD) (Castelli, 1988), a leading cause of mortality worldwide. Because of the well-established association between lipid levels and CVD, lipid profiles are considered key clinical indicators of this disease. In Taiwan, serum lipid levels are routinely measured as part of monitoring for many chronic diseases. A lipid profile comprises measurements of total cholesterol (TC), high-density lipoprotein cholesterol (HDL-C), low-density lipoprotein cholesterol (LDL-C), and triglycerides (TGs). Identifying biological factors that affect lipid metabolism and its association with CVD pathophysiology may facilitate the development of novel therapeutic strategies for this disease.

Blood lipid levels are heritable traits, and known genetic factors account for approximately 10%–15% of the total variance in these traits (Pilia et al., 2006). Familial hypercholesterolemia (FH) is a common inherited disorder characterized by an abnormally high serum LDL-C level. Mutations in the gene encoding the LDL receptor are the primary cause of FH; however, mutations in other genes involved in LDL metabolism, such as *APOB* and *PCSK9*, can also cause FH (Cuchel et al., 2014). Familial combined hyperlipidemia is an oligogenic primary lipid disorder characterized by fluctuating serum lipid levels (Bello-Chavolla et al., 2018). Therefore, genetic associations between blood lipid levels and lipid-related diseases should be investigated to inform lipid management strategies and develop targeted therapies.

A genome-wide association study (GWAS) is a powerful approach for identifying genetic variants associated with specific traits or diseases across the human genome (Chen et al., 2024a; Chen et al., 2024b; Chen et al., 2024c; Liu et al., 2023). The first large-scale GWAS of lipid traits involved 2,800 individuals and revealed that *CETP* and *APOE* were associated with HDL-C and LDL-C, respectively, thereby confirming the findings of other genetic studies (Saxena et al., 2007). Moreover, a meta-analysis of 46 cohorts involving approximately 100,000 individuals identified 31, 22, 16, and 39 loci associated with HDL-C, LDL-C, TGs, and TC, respectively (Teslovich et al., 2010). Although most GWASs have focused on European populations, new loci have also been identified in East Asian (EAS) populations (Kim et al., 2011). Progressively larger GWASs and trans-ancestry meta-analyses have identified more than 900 loci associated with serum lipid traits (Klarin et al., 2018; Graham et al., 2021).

The polygenic risk score (PRS) is a quantitative measure of an individual’s genetic susceptibility to specific traits or diseases. The PRS is calculated using genetic variants from across the human genome. Because the PRS can help estimate the risk of various conditions, it has been widely used in personalized medicine and for risk assessment (Lin et al., 2023; Chen et al., 2024d; Huang et al., 2024; Liao and Tsai, 2013). PRSs for lipid traits are currently used to study dyslipidemia and have also been occasionally applied in clinical practice (Dron et al., 2020; Wand et al., 2021). However, the generalizability of existing lipid PRS models to the unique genetic architecture of the Taiwanese Han population remains unclear, particularly regarding the effects of trans-ancestry weight dilution, in which effect-size weights derived from one ancestral population may exhibit low predictive performance in another population. To address this critical gap, we performed large-scale GWASs to identify population-specific genetic signals and establish a robust Taiwan-specific PRS framework for predicting blood lipid levels and CVD. Additionally, we conducted a trans-ancestry meta-analysis to evaluate model performance across diverse genetic backgrounds and assess the clinical utility and applicability of population-specific genomic risk assessments.

## 2 Materials and methods

### 2.1 Study cohort and data

This study used electronic medical records from China Medical University Hospital (CMUH). Eligible patients were identified on the basis of their lipid profile results, namely, TC, HDL-C, LDL-C, and TG levels and the ratio of LDL-C to TC (LDL-C/TC ratio). Because multiple records were available for each patient, we selected the maximum TC, LDL-C, and TG levels and the minimum HDL-C level as representative measurements. We calculated the LDL-C/TC ratio by using the TC value obtained from the same blood sample as the maximum LDL-C value. For female patients, we excluded measurements obtained during pregnancy from the analysis. The study protocol was approved by the Research Ethics Committee of CMUH (approval numbers: CMUH110-REC3-005 and CMUH111-REC1-176). Informed consent was obtained from all included patients. Moreover, to independently validate the generalizability of our findings, we obtained genotypic and phenotypic data from 120,140 participants in the Taiwan Biobank (TWB), a large-scale community-based prospective cohort. Access to and use of TWB data were approved by the Ethics Governance Committee of the TWB (Application Project Number: TWBR11208-04). All TWB participants provided written informed consent before data collection, and all study procedures were conducted in accordance with the Declaration of Helsinki.

### 2.2 Genotyping

Venous blood samples were collected and stored in ethylenediaminetetraacetate tubes. Genomic DNA was extracted from 200 µL of peripheral blood by using the MagCore Genomic DNA Whole Blood Kit (RBC Bioscience, New Taipei City, Taiwan) in accordance with the manufacturer’s instructions. Genomic data of the Taiwanese Han population were obtained using the Affymetrix Axiom genotyping platform, specifically the Axiom Taiwan Precision Medicine customized single-nucleotide polymorphism (SNP) array (Thermo Fisher Scientific, Santa Clara, CA, United States). This array covers 714,457 SNPs across the human genome. We analyzed the data by using PLINK 1.9 and excluded samples and SNPs with missing rates. Furthermore, we excluded variants that failed to meet the Hardy–Weinberg equilibrium criterion (*p* < 1e^−6^) or had a minor allele frequency of <1e^−4^. To control for population stratification and ensure genetic homogeneity, an in-sample principal component analysis (PCA) was subsequently performed on our QC-passed CMUH dataset, following a validated pipeline from our previous institutional study characterizing this cohort. Non-Taiwanese Han outliers who deviated significantly from the central genomic cluster based on standard deviation thresholds across the first two PCs were rigorously filtered and excluded. The first 10 principal components (PC1–10) were retained to capture subtle population substructures, and a representative PCA plot plotted alongside 1000 Genomes East Asian (EAS) reference samples has been previously established and detailed in our previous study (Liu et al., 2025) (see their Figure 3). Genomic data were phased using SHAPEIT4, and genotype imputation was performed using Beagle5.2, which is known for its higher efficacy and accuracy compared with other imputation tools. The imputed data were filtered on the basis of the following criteria: an alternate allele dosage *r* ^2^ threshold of <0.3 and a genotype posterior probability of <0.9 (Liu et al., 2021).

### 2.3 GWASs

To identify genetic variants associated with lipid traits, including TC, HDL-C, LDL-C, and TG levels and the LDL-C/TC ratio, we obtained summary statistics by using PLINK 1.9. Familial relationships were determined using the KINSHIP function in PLINK 2.0, and second-degree relatives were excluded. For GWAS quality control, we excluded heterozygous outliers that were more than 5 standard deviations from the mean and principal component analysis outliers that were more than three interquartile ranges from the median. Linear regression analysis was performed to examine associations between genetic variants and lipid traits after adjustment for several covariates, including sex, age, and principal components. To prevent collinearity-induced overestimation, we selected only the most significant variant. Variants for which the *p* value was <1 × 10^−5^ or, under a more stringent threshold, <5 × 10^−8^ were considered to be significantly associated with blood lipid traits. For data visualization, Manhattan and quantile–quantile plots were generated using the R packages “qqman” and “topr.”

### 2.4 PRS analysis

To calculate PRSs for blood lipid traits, we divided the study cohort into base and replication groups. The base group comprised 80% of the included patients, whereas the replication group comprised the remaining 20%. We used data from the base group to explore the associations between genetic variants and blood lipid levels. PRSs were calculated using Polygenic Risk Scores-Continuous Shrinkage (PRS-CS; version 1.0.0; https://github.com/getian107/PRScs/releases), a Bayesian regression framework that uses external linkage disequilibrium (LD) information for the polygenic prediction of disease risk (Ge et al., 2019). We used the EAS LD reference panel provided by the PRS-CS authors, which was derived from the 1000 Genomes Project Phase 3 dataset (ldblk_1kg_eas). Before PRS calculation, GWAS summary statistics were harmonized, and ambiguous SNPs or poorly imputed variants were excluded during preprocessing. The target dataset consisted of quality-controlled imputed genotype data generated using the Taiwan Precision Medicine Initiative array platform. PRS-CS was executed chromosome-wise for all 22 autosomes by using the default Gibbs sampling framework. Because our discovery dataset included more than 100,000 individuals, the global shrinkage parameter (ψ) was automatically estimated from the data by using the fully Bayesian approach implemented in PRS-CS. Default Markov chain Monte Carlo settings were applied, including 1,000 total iterations, 500 burn-in iterations, and a thinning factor of 5. The estimated posterior SNP weights were subsequently combined across all autosomes, and then individual PRS values were calculated using the--score function in PLINK v1.9. The calculated PRS values were then used to construct PRS models for predicting blood lipid levels and CVD. Finally, we evaluated the predictive performance of the PRS models by using receiver operating characteristic (ROC) curves and corresponding area under the curve (AUC) values. These analyses were performed using SPSS (version 22; IBM Corporation, Armonk, NY, United States).

### 2.5 Meta-analysis

We conducted a trans-ancestry meta-analysis using METAL (Willer et al., 2010). We meta-analyzed the summary statistics of the CMUH cohort with those of five genetic ancestry cohorts from the Global Lipids Genetics Consortium (GLGC): Admixed African or African, EAS, European, Hispanic, and South Asian. To ensure consistency between the hg19/GRCh37 and hg38/GRCh38 reference genome assemblies, we converted variants from both the GLGC datasets and our study into the rsID format by using dbSNP (version 153) before performing the meta-analysis. This step facilitated variant matching across datasets and reduced discrepancies resulting from differences in reference genome assemblies.

### 2.6 Phenome-wide association study

A total of 97,735,180 diagnostic codes from the International Classification of Diseases, Ninth Revision, and International Classification of Diseases, Tenth Revision, were consolidated into 1,791 phecodes. We examined the association between each phecode and the PRS by using logistic regression implemented in the “PheWAS” package in R (Carroll et al., 2014). To control for Type I errors across multiple comparisons, we applied two adjustment methods for multiple testing: Bonferroni correction was used to define a stringent genome-wide significance threshold, and the false discovery rate approach was used to control for potential false positives under a broader exploratory threshold.

### 2.7 Statistical analysis

Baseline continuous and categorical variables across the genotype groups were analyzed using Student’s t-test, a chi-square test, or Fisher’s exact test, as appropriate. Between-group comparisons were performed using one-way analysis of variance, followed by Tukey’s *post hoc* test. A two-sided *p* value of <0.05 was considered statistically significant. All statistical analyses were performed using SPSS (version 22) and R (version 4.1.0).

## 3 Results

### 3.1 GWAS results

**FIGURE 1:**
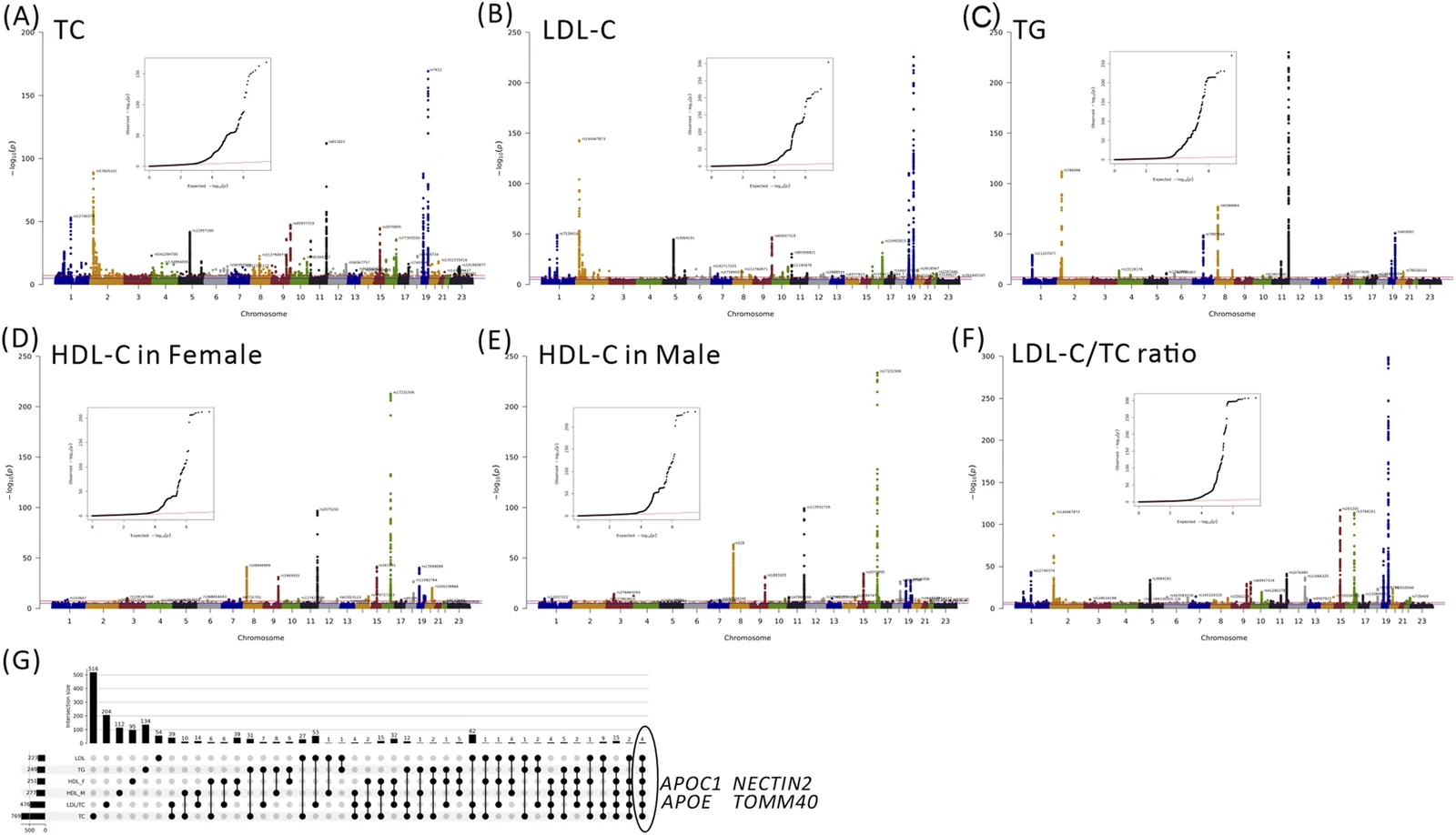
Manhattan and quantile–quantile plots for blood lipid levels and Venn diagram depicting significant genes across lipid traits. **(A–F)** The Manhattan plot depicts the peaks of single-nucleotide polymorphisms that exceeded the genome-wide significance thresholds. The blue line indicates a threshold of 1 × 10^−5^. The red line indicates a threshold of 5 × 10^−8^. The quantile–quantile plot depicts the agreement between the observed and expected *p* values. The red line represents the expected values. **(G)** The Venn diagram presents the numbers of significant genes identified for TC levels (769 genes), LDL-C levels (224 genes), TG levels (250 genes), HDL-C levels (252 genes) in female patients, HDL-C levels (278 genes) in male patients, and the LDL-C/TC ratio (476 genes). An intersection analysis across the traits revealed four genes common to all traits: *APOC1*, *APOE*, *NECTIN2*, and *TOMM40*. Abbreviations: TC, total cholesterol; LDL-C, low-density lipoprotein cholesterol; TG, triglyceride; HDL-C, high-density lipoprotein cholesterol.

Eligible patients were identified from the CMUH genetic biobank, which was established in 2018. This biobank integrates patients’ genotyping data with their electronic medical records. To account for sex-based differences in standard HDL-C levels, we performed separate analyses for men and women. The GWAS workflow is illustrated in Supplementary Figure S1. GWAS results for each blood lipid trait were visualized using Manhattan and quantile–quantile plots (Figures 1A–E). These plots revealed associations between genetic variants and lipid traits. To further examine genetic variants associated with cholesterol composition, we performed a GWAS of the LDL-C/TC ratio (Figure 1F). We compared genes associated with the six lipid traits and determined that four genes, namely, *APOC1*, *APOE*, *NECTIN2*, and *TOMM40*, significantly varied across the traits (Figure 1G). The top 10 most significantly associated genes for each trait are listed in Table 1. Supplementary Tables S1–S6 present comprehensive lists of genes associated with each lipid trait. Furthermore, we compared the 30 genes most significantly associated with each lipid trait with those reported in the GWAS catalog and identified novel associations, which are summarized in Table 2.

**TABLE 1:** Top 10 genome-wide significant genes identified for each of the six lipid traits in the GWAS analysis.

Chr	Pos	Ref	Alt	rsID	Nearest gene	-log 10(p)
TC						
19	44,908,822	C	T	s7412	APOE	169.155
11	116,791,863	C	T	s651821	APOA5	112.104
2	21,024,193	T	A	s57825321	APOB	88.782
19	11,116,900	C	T	s730882109	LDLR	87.901
1	109,274,968	G	T	s12740374	CELSR2	53.033
2	22,106,856	C	A	s150365850	AC096570.1	50.021
9	133,264,504	G	GAAACTGCC	s60937319	ABO	47.376
15	58,431,740	G	A	s2070895	ALDH1A2, LIPC	44.519
5	75,312,907	C	T	s11957260	AC008897.2	41.609
9	104,902,020	C	T	s1883025	ABCA1	36.053
LDL-C						
19	44,881,075	GC	G	s34994196	NECTIN2	Infinity
2	21,006,289	G	A	s144467873	APOB	142.956
19	11,116,900	C	T	s730882109	LDLR	110.332
1	109,274,570	A	G	s7528419	CELSR2	49.002
9	133,264,504	G	GAAACTGCC	s60937319	ABO	46.721
5	75,343,719	CTTGTA	C	s3064191	HMGCR	44.712
2	22,931,058	A	G	s1390547021	RN7SKP27	42.752
16	72,129,795	A	G	s10492815	PMFBP1	41.816
11	5,226,762	CAAAG	C	s80356821	HBB	30.295
2	43,824,071	C	T	s119480069	ABCG5	29.422
TG						
11	116,791,863	C	T	s651821	APOA5	Infinity
2	27,518,205	C	G	s780096	GCKR	111.863
8	20,005,847	T	C	s6586884	LPL	76.96
19	44,912,921	G	T	s483082	AC011481.3	50.817
7	73,621,527	T	C	s7800944	MLXIPL	48.439
1	62,511,636	C	T	s11207977	DOCK7	29.227
19	19,268,740	C	T	s58542926	AC138430.1, TM6SF2	23.321
11	61,842,278	C	T	s174583	FADS2	22.887
2	21,029,662	G	A	s13306194	APOB	21.765
8	125,487,789	C	G	s28601761	AC091114.1	14.342
HDL-C-female						
16	56,960,616	C	T	s17231,506	CETP	212.88
11	116,790,676	C	A	s2075291	APOA5	96.778
15	58,387,979	T	C	s261291	ALDH1A2	41.386
8	19,982,830	G	A	s58946909	LPL	41.242
19	11,233,119	A	G	s17699089	DOCK6	40.326
9	104,902,020	C	T	s1883025	ABCA1	31.126
18	49,593,209	A	G	s11082764	LIPG	26.807
16	55,993,350	A	T	s1340273649	AC040168.1	23.548
20	45,912,201	CCCT	C	s200238866	PLTP	20.447
19	44,908,684	T	C	s429358	APOE	12.829
HDL-C-male						
16	56,960,616	C	T	s17231,506	CETP	233.867
11	116,779,922	C	T	s113932726	ZPR1	99.165
8	19,962,213	C	G	s328	LPL	63.468
15	58,431,740	G	A	s2070895	ALDH1A2, LIPC	34.686
9	104,902,020	C	T	s1883025	ABCA1	31.528
19	44,908,684	T	C	s429358	APOE	27.693
19	11,236,981	C	T	s737338	DOCK6	27.511
18	49,592,028	T	C	s9958734	LIPG	26.562
3	52,511,910	C	T	s376663293	STAB1	14.408
3	51,486,571	C	T	s373561619	DCAF1	13.547
LDL-C/TC ratio						
19	44,875,803	A	C	rs387976	NECTIN2	Infinity
15	58,387,979	T	C	rs261291	ALDH1A2	117.05
16	56,959,412	C	A	rs3764261	CETP	113.661
2	21,006,289	G	A	rs144467873	APOB	113.125
19	11,116,900	C	T	rs730882109	LDLR	70.886
2	22,106,856	C	A	rs150365850	AC096570.1	60.169
1	109,274,968	G	T	rs12740374	CELSR2	43.297
11	116,901,725	C	T	rs1076485	SIK3	41.066
12	112,492,671	T	C	rs11066325	PTPN11	36.674
5	75,343,719	CTTGTA	C	rs3064191	HMGCR	33.814

Chr, chromosome; Pos, position; Ref, reference; Alt, alternative; TC, total cholesterol; LDL-C, low-density lipoprotein cholesterol; TG, triglyceride; HDL-C, high-density lipoprotein cholesterol.

**TABLE 2:** Novel genes identified in the top 30 GWAS signals for each lipid trait after comparison with the GWAS catalog.

Novel genes	GWAS catalog reported	Previous studies reported functions	PMID
TC			
EDEM3		Associated with TG	32213464
ESRRG		Involved in lipid metabolism	19949424
NPRL3			
PHACTR3	Fatty acid measurement		
RPS27P1			
STAP1		Involved in hypercholesterolemia	25035151
LDL-C			
DST			
GET3			
GLMN	Total cholesterol measurement		
LIMS2			
NPRL3			
RNU6-1140P			
STK39			
TG			
ATXN1L	TC, HDL, LDL		
ITGA2B			
HDL-C-female			
C3orf18			
DCP1A	LDL		
DOCK3		Involved in hyperglycemia and increased fat mass	37742307
FBXW12			
MMP2		Involved in lipid metabolism	26567374
NUFIP1P1			
PHACTR1			
HDL-C-male			
AGXT			
CISH		Involved in lipid metabolism and lipid accumulation in chicken and mice	33519913, 34516753
DCAF1	Lipoprotein A		
FBXW12			
HBB	TC, LDL		
INTS6		Involved in lipid accumulation	38981571
LSG1			
LDL-C/TC ratio			
ZNF415			
CRHBP			
WDR25			

TC, total cholesterol; LDL-C, low-density lipoprotein cholesterol; TG, triglyceride; HDL-C-Female, high-density lipoprotein cholesterol in female patients; HDL-C-Male, high-density lipoprotein cholesterol in male patients.

### 3.2 PRS models of blood lipid traits

**FIGURE 2:**
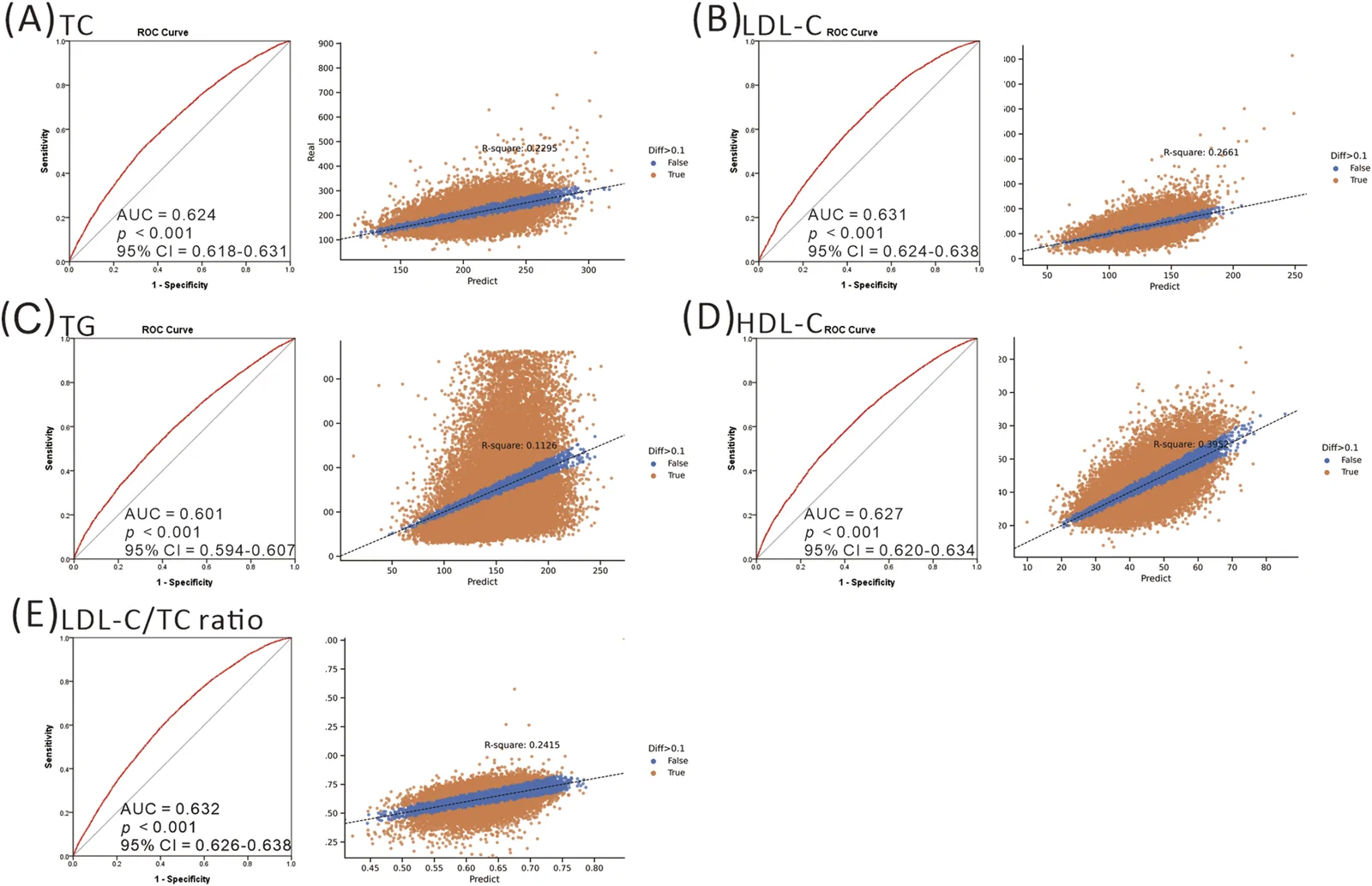
Performance of the PRS model in predicting blood lipid levels. **(A–E)** The left panels present the results of AUC analyses performed to evaluate the accuracy of our PRS models in predicting dyslipidemia in the replication group. The right panels present the correlations between model-predicted and measured blood lipid levels, indicating model performance in predicting blood lipid levels. Diff > 0.1 indicates that the difference between the predicted and measured values [abs (actual − predicted)/actual] exceeded 0.1%. Orange dots represent samples with Diff > 0.1, whereas blue dots represent samples with Diff < 0.1. Abbreviations: PRS, polygenic risk score; AUC, area under the curve; ROC curve, receiver operating characteristic curve.

On the basis of the GWAS results, we developed PRS models to evaluate the cumulative effects of multiple genetic variants associated with lipid traits. PRS models are widely used to determine genetic risk, predict disease susceptibility, refine diagnostics, develop personalized treatment regimens, and advance therapeutic development. We developed separate PRS models for TC, LDL-C, TG, and HDL-C levels as well as for the LDL-C/TC ratio. We then compared lipid levels and the LDL/TC ratio predicted using these models with the corresponding observed measurements. Correlation analyses yielded *r* ^*2*^ values of 0.2295, 0.2661, 0.2614, and 0.3952 for TC (Figure 2A), LDL-C (Figure 2B), TG (Figure 2C), and HDL-C (Figure 2D) levels, respectively. An *r* ^2^ value of 0.2415 was observed for the LDL-C/TC ratio (Figure 2E). To evaluate the accuracy of the PRS model–based classification, we divided the cohort into normal and dyslipidemia groups on the basis of reference lipid thresholds defined by Taiwan’s Ministry of Health and Welfare. ROC curve analysis yielded AUC values of 0.624, 0.631, 0.601, 0.649 and 0.632 for TC levels (Figure 2A), LDL-C levels (Figure 2B), TG levels (Figure 2C), HDL-C levels (Figure 2D), and the LDL-C/TC ratio (Figure 2E), respectively. To examine the statistical robustness of our PRS models with respect to clinical treatment noise and individual variation within this hospital-based cohort, we conducted a sensitivity analysis. Specifically, we examined the correlation between PRSs derived from representative extreme lipid values and the participants’ mean clinical lipid levels. As displayed in Supplementary Figure S2, the PRSs derived from the extreme lipid values were significantly associated with both extreme and mean lipid measurements for all four lipid traits (*p* < 0.001); the *r* ^*2*^ values for TC, LDL-C, HDL-C, and TG levels were 0.175, 0.205, 0.393, and 0.230, respectively.

To further evaluate the generalizability of the Taiwan-specific PRS and rule out potential overfitting, we performed an independent external validation by using data from the TWB (*N* = 120,140). Specifically, we calculated the PRSs for the TWB participants by using weights derived from the CMUH cohort and evaluated the correlation between estimated and observed lipid levels in the TWB cohort. As illustrated in Supplementary Figure S3, the *r* ^2^ values for TC, LDL-C, HDL-C, and TG levels were 0.106, 0.094, 0.234, and 0.087, respectively (all *p* < 0.001).

### 3.3 PheWAS results

**FIGURE 3:**
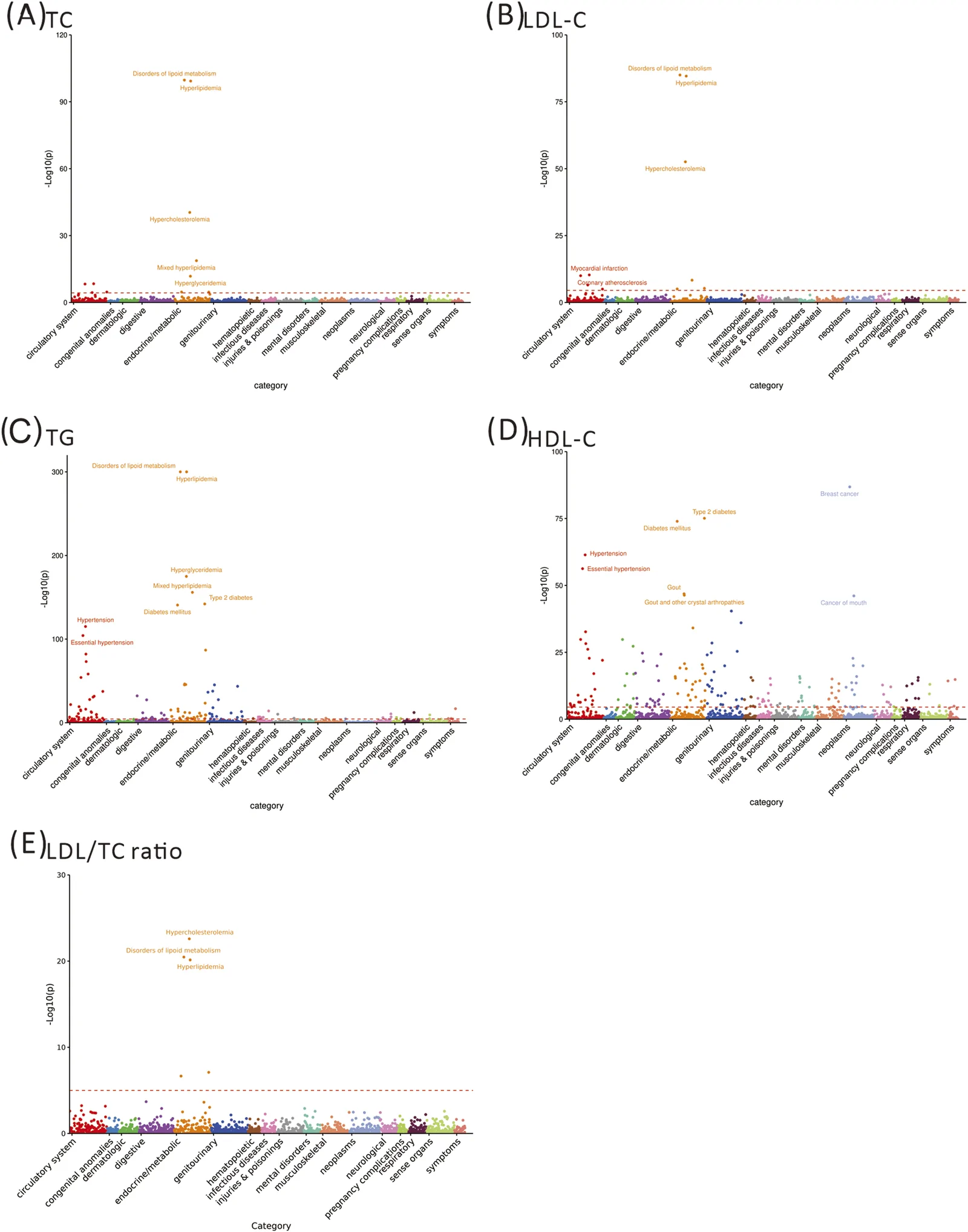
Manhattan plots illustrating the associations of phecodes with the PRSs of various blood lipid levels. The plots depict the associations of 1,792 phecodes with PRSs based on TC levels **(A)**, LDL-C levels **(B)**, TG levels **(C)**, HDL-C levels **(D)**, and the LDL-C/TC ratio **(E)**. The phecodes were grouped into different disease categories and are presented using various colors in the Manhattan plots. The dashed red line indicates a threshold of 0.00003, corresponding to the *p* value adjusted for multiple comparisons by using false discovery rate correction. Abbreviations: PRS, polygenic risk score; TC, total cholesterol; LDL-C, low-density lipoprotein cholesterol; TG, triglyceride; HDL-C, high-density lipoprotein cholesterol.

We conducted a PheWAS by using the PRSs derived for the four lipid traits. Individuals included in the GWAS analyses were excluded from the PheWAS analysis. Associations between lipid PRSs and 1,791 phecodes were visualized using a Manhattan plot (Figure 3). The top 10 most significant phenotypes for each trait are listed in Table 3. Disorders of lipoid metabolism and hyperlipidemia were the top two phenotypes most significantly associated with TC levels, LDL-C levels, TG levels, and the LDL-C/TC ratio, whereas significant but less prominent associations were also observed for HDL-C levels (Figure 3; Supplementary Tables S7–S11).

**TABLE 3:** PheWAS results for PRSs for each lipid trait.

Phecode	Description	Group	Beta	SE	OR	p	n_total	n_cases	n_controls	Bonferroni	fdr
TC											
272	Disorders of lipoid metabolism	Endocrine/metabolic	1.041935	0.048971	2.834698	1.87E-100	181,143	10,177	170,966	True	True
272.1	Hyperlipidemia	Endocrine/metabolic	1.04102	0.049033	2.832103	4.94E-100	181,115	10,149	170,966	True	True
272.11	Hypercholesterolemia	Endocrine/metabolic	1.09766	0.08173	2.997143	4.02E-41	174,477	3,511	170,966	True	True
272.13	Mixed hyperlipidemia	Endocrine/metabolic	1.1879	0.131609	3.280187	1.78E-19	172,301	1,335	170,966	True	True
272.12	Hyperglyceridemia	Endocrine/metabolic	1.436605	0.203432	4.206392	1.64E-12	171,523	557	170,966	True	True
411.2	Myocardial infarction	Circulatory system	0.820924	0.139483	2.272598	3.97E-09	175,761	1,183	174,578	True	True
411.4	Coronary atherosclerosis	Circulatory system	0.523143	0.089659	1.687323	5.38E-09	177,462	2,884	174,578	True	True
411.1	Unstable angina (intermediate coronary syndrome)	Circulatory system	0.432438	0.101431	1.54101	2.01E-05	176,822	2,244	174,578	True	True
250	Diabetes mellitus	Endocrine/metabolic	0.185116	0.043472	1.203358	2.06E-05	179,262	12,985	166,277	True	True
250.2	Type 2 diabetes	Endocrine/metabolic	0.185534	0.043684	1.203861	2.17E-05	179,126	12,849	166,277	True	True
LDL-C											
272	Disorders of lipoid metabolism	Endocrine/metabolic	0.948752	0.048347	2.582484	9.69E-86	198,688	11,385	187,303	True	True
272.1	Hyperlipidemia	Endocrine/metabolic	0.947615	0.048408	2.57955	2.50E-85	198,657	11,354	187,303	True	True
272.11	Hypercholesterolemia	Endocrine/metabolic	1.243525	0.080911	3.467817	2.64E-53	191,273	3,970	187,303	True	True
411.2	Myocardial infarction	Circulatory system	0.939552	0.142988	2.558836	5.00E-11	193,162	1,235	191,927	True	True
411.4	Coronary atherosclerosis	Circulatory system	0.585452	0.090497	1.795802	9.84E-11	194,984	3,057	191,927	True	True
272.13	Mixed hyperlipidemia	Endocrine/metabolic	0.744697	0.127095	2.105804	4.65E-09	188,851	1,548	187,303	True	True
411	Ischemic heart disease	Circulatory system	0.32959	0.064228	1.390397	2.87E-07	198,004	6,077	191,927	True	True
250.2	Type 2 diabetes	Endocrine/metabolic	0.191712	0.042032	1.211321	5.09E-06	196,857	14,760	182,097	True	True
250	Diabetes mellitus	Endocrine/metabolic	0.186129	0.041827	1.204577	8.59E-06	197,010	14,913	182,097	True	True
411.1	Unstable angina (intermediate coronary syndrome)	Circulatory system	0.449254	0.101617	1.567142	9.82E-06	194,326	2,399	191,927	True	True
TG											
272	Disorders of lipoid metabolism	Endocrine/metabolic	1.424323	0.036962	4.155042	0	186,280	14,270	172,010	True	True
272.1	Hyperlipidemia	Endocrine/metabolic	1.426593	0.037006	4.164488	0	186,242	14,232	172,010	True	True
272.12	Hyperglyceridemia	Endocrine/metabolic	3.2943	0.116548	26.95855	9.14E-176	173,300	1,290	172,010	True	True
272.13	Mixed hyperlipidemia	Endocrine/metabolic	2.252391	0.084471	9.510445	1.21E-156	174,508	2,498	172,010	True	True
250.2	Type 2 diabetes	Endocrine/metabolic	0.88662	0.034853	2.426914	9.46E-143	184,497	16,223	168,274	True	True
250	Diabetes mellitus	Endocrine/metabolic	0.878941	0.034711	2.408347	1.85E-141	184,646	16,372	168,274	True	True
401	Hypertension	Circulatory system	0.653296	0.028562	1.921865	8.59E-116	182,420	25,804	156,616	True	True
401.1	Essential hypertension	Circulatory system	0.664491	0.030535	1.943501	5.38E-105	178,731	22,115	156,616	True	True
250.22	Type 2 diabetes with renal manifestations	Endocrine/metabolic	1.543915	0.077841	4.682889	1.51E-87	171,235	2,961	168,274	True	True
401.2	Hypertensive heart and/or renal disease	Circulatory system	0.73537	0.038143	2.086253	8.05E-83	170,092	13,476	156,616	True	True
HDL-C											
174	Breast cancer	Neoplasms	0.914871	0.046126	2.496454	1.51E-87	193,090	5,095	187,995	True	True
250.2	Type 2 diabetes	Endocrine/metabolic	−0.51549	0.027978	0.597209	8.27E-76	196,908	15,241	181,667	True	True
250	Diabetes mellitus	Endocrine/metabolic	−0.50924	0.027852	0.600953	1.12E-74	197,052	15,385	181,667	True	True
401	Hypertension	Circulatory system	−0.35882	0.02157	0.698503	3.91E-62	194,463	27,353	167,110	True	True
401.1	Essential hypertension	Circulatory system	−0.36753	0.023103	0.692442	5.55E-57	190,466	23,356	167,110	True	True
274.1	Gout	Endocrine/metabolic	−0.71488	0.049328	0.489252	1.36E-47	200,409	4,660	195,749	True	True
274	Gout and other crystal arthropathies	Endocrine/metabolic	−0.70828	0.049153	0.492493	4.50E-47	200,442	4,693	195,749	True	True
145	Cancer of mouth	Neoplasms	−1.08201	0.075299	0.338913	8.03E-47	198,823	1999	196,824	True	True
613	Other nonmalignant breast conditions	Genitourinary	0.806797	0.060056	2.240719	3.82E-41	197,631	2,961	194,670	True	True
594	Urinary calculus	Genitourinary	−0.49459	0.039054	0.609824	9.35E-37	195,925	7,496	188,429	True	True
LDL-C/TC ratio											
272.11	Hypercholesterolemia	Endocrine/metabolic	0.72222	0.072607	2.058999	2.60E-23	177,978	3,523	174,455	True	True
272	Disorders of lipoid metabolism	Endocrine/metabolic	0.400052	0.042336	1.491903	3.40E-21	184,932	10,477	174,455	True	True
272.1	Hyperlipidemia	Endocrine/metabolic	0.397297	0.0424	1.487797	7.23E-21	184,897	10,442	174,455	True	True
250.2	Type 2 diabetes	Endocrine/metabolic	0.197471	0.036795	1.218318	8.01E-08	182,846	13,949	168,897	True	True
250	Diabetes mellitus	Endocrine/metabolic	0.189805	0.036619	1.209014	2.18E-07	182,985	14,088	168,897	True	True
577.2	Chronic pancreatitis	Digestive	−0.79988	0.215355	0.449382	0.00020381	186,415	347	186,068	False	True
259.4	Precocious sexual development and puberty NEC	Endocrine/metabolic	0.324321	0.088181	1.383092	0.00023515	180,136	2,301	177,835	False	True
433	Cerebrovascular disease	Circulatory system	0.200723	0.058429	1.222286	0.00059188	184,020	5,271	178,749	False	False
433.31	Transient cerebral ischemia	Circulatory system	0.444866	0.130989	1.56028	0.00068328	179,794	1,045	178,749	False	False
250.22	Type 2 diabetes with renal manifestations	Endocrine/metabolic	0.331149	0.099926	1.392568	0.00091993	170,689	1792	168,897	False	False

SE, standard error; OR, odds ratio; p, *p* value; bonferroni, Bonferroni-corrected *p* value; fdr, false discovery rate-adjusted *p* value; n_total, total number of participants; n_cases, number of cases; n_controls, number of controls.

### 3.4 Meta-analysis results

**FIGURE 4:**
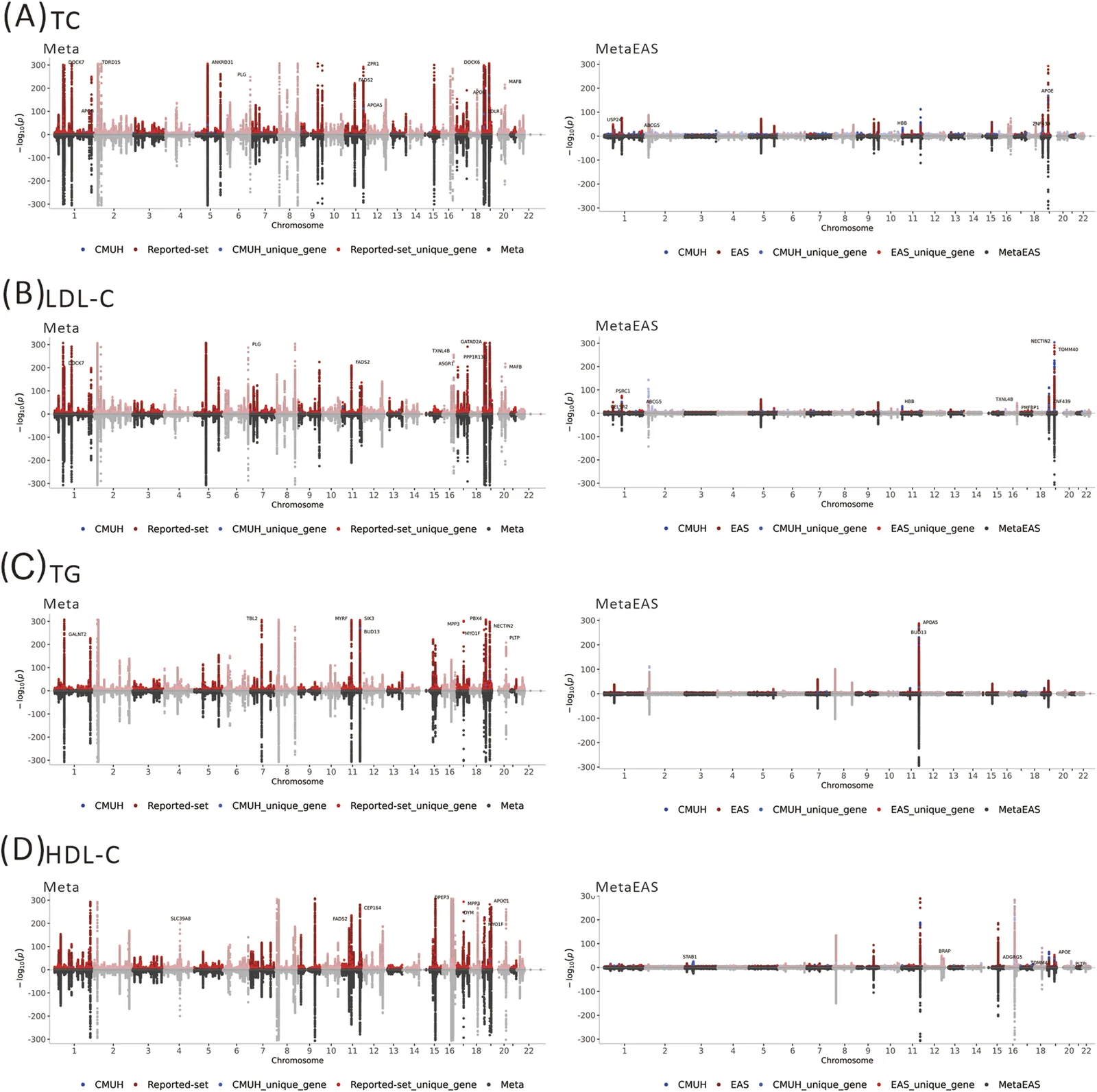
Manhattan plots depicting the meta-analysis results for various blood lipid levels. The left panels present the meta-analysis results for TC **(A)**, LDL-C **(B)**, TG **(C)**, and HDL-C **(D)** levels. The meta-analysis was performed by combining the CMUH cohort with five ancestry cohorts. The right panels present the results of a meta-analysis that was performed by combining the CMUH and EAS cohorts. In each Manhattan plot, the upper section presents the GWAS results for the CMUH cohort and the five genetic ancestry cohorts (left panels) or the EAS cohort (right panels). The lower section presents the corresponding meta-analysis results. GWAS results for the CMUH cohort are presented in blue, whereas those for the five ancestry cohorts or the EAS cohort are presented in red. The meta-analysis results are presented in gray. Abbreviations: TC, total cholesterol; LDL-C, low-density lipoprotein cholesterol; TG, triglyceride; HDL-C, high-density lipoprotein cholesterol; CMUH, China Medical University Hospital; EAS, East Asian; GWAS, genome-wide association study.

**FIGURE 5:**
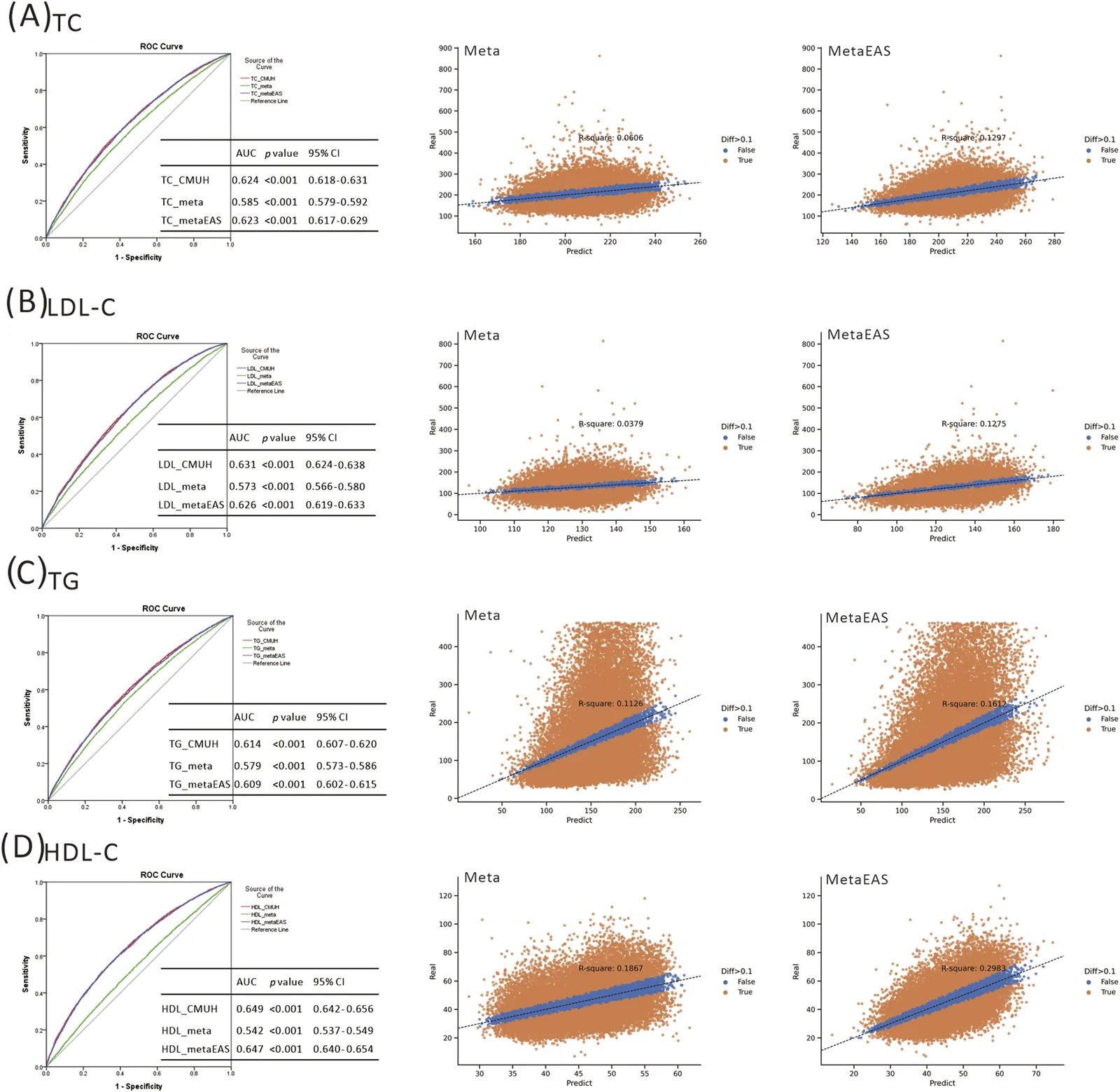
Performance of PRS models in predicting blood lipid levels in the meta-analysis. **(A–D)** The left panels present the results of AUC analyses performed to evaluate the accuracy of the PRS models in predicting dyslipidemia in the replication group. Lipid_CMUH indicates the PRS model developed using only the CMUH cohort. Lipid_meta indicates the PRS model developed through a meta-analysis of the CMUH and five ancestry cohorts. Lipid_metaEAS indicates the PRS model developed through a meta-analysis of the CMUH and EAS cohorts. The right panels present the correlations between model-predicted and measured blood lipid levels, indicating model performance in predicting blood lipid levels. Diff > 0.1 indicates that the difference between the predicted and measured values [abs (actual − predicted)/actual] exceeded 0.1%. Orange dots represent samples with Diff > 0.1, whereas blue dots represent samples with Diff < 0.1. Abbreviations: PRS, polygenic risk score; AUC, area under the curve; ROC curve, receiver operating characteristic curve; CI, confidence interval; CMUH, China Medical University Hospital; EAS, East Asian.

We performed a trans-ancestry meta-analysis by using GWAS summary statistics from the GLGC dataset. This dataset includes more than 1.65 million individuals from five genetic ancestry groups: Admixed African or African (*n* = 99,432), EAS (*n* = 146,492), European (*n* = 1,320,016), Hispanic (*n* = 48,057), and South Asian (*n* = 40,963) (Graham et al., 2021). We conducted the meta-analysis by combining the CMUH cohort with these five cohorts to identify trans-ancestry genetic variants associated with blood lipid traits. The meta-analysis results significantly differed between the GLGC and CMUH cohorts (Figure 4). Given the genetic similarity between the CMUH and EAS cohorts, we conducted an additional meta-analysis by combining these two cohorts. The results were consistent with those obtained from the CMUH cohort alone, and stronger statistical significance was observed for the associated genes (Figure 4). We developed PRS models through meta-analyses of the GLGC–CMUH and EAS–CMUH cohorts. We used these models to investigate the correlations between the predicted and measured lipid levels, and we evaluated model performance by using AUC values. In the PRS model developed through a meta-analysis of the GLGC–CMUH cohorts, the *r* ^*2*^ values for the correlations between the predicted and measured lipid levels were 0.0606, 0.0379, 0.1126, and 0.1867 for TC (Figure 5A), LDL-C (Figure 5B), TG (Figure 5C), and HDL-C (Figure 5D) levels, respectively. The corresponding AUC values of the PRS models based on the TC (Figure 5A), LDL-C (Figure 5B), TG (Figure 5C), and HDL-C (Figure 5D) levels were 0.585, 0.573, 0.579, and 0.542, respectively. In the PRS model developed through a meta-analysis of the EAS–CMUH cohorts, the *r* ^*2*^ values for the correlations between the predicted and measured lipid levels were 0.1297, 0.1275, 0.1612, and 0.2983 for TC, LDL-C, TG, and HDL-C levels, respectively (Figure 5). The corresponding AUC values of the PRS models based on TC, LDL-C, TG, and HDL-C levels were 0.623, 0.626, 0.609, and 0.647, respectively (Figure 5). To systematically evaluate differences in genetic architecture between the Taiwanese population and other ancestry groups, we performed a cross-ancestry heterogeneity analysis by comparing our CMUH summary statistics with global meta-analysis data. As presented in Supplementary Table S12, a substantial number of genome-wide significant loci exhibited opposite directions of association between the CMUH cohort and the five global populations. Specifically, we identified 4,478, 2,696, 4,514, and 4,418 discordant loci for TC, LDL-C, HDL-C, and TG levels, respectively. Among these discordant loci, 25.6%, 24.3%, 38.7%, and 15.4% for TC, LDL-C, HDL-C, and TG levels, respectively, exhibited statistical heterogeneity (I^2^ > 50%).

### 3.5 PRS model–based disease prediction

**FIGURE 6:**
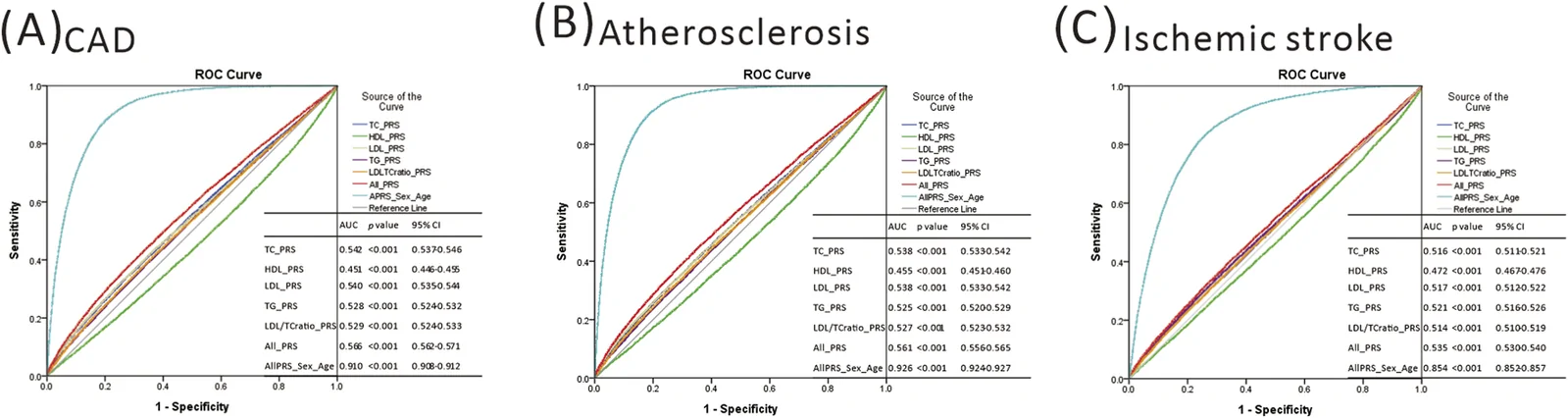
Prediction of lipid-related diseases on the basis of the PRSs for various blood lipids. AUC analyses were performed to evaluate the performance of PRS models based on TC levels, LDL-C levels, TG levels, HDL-C levels, and the LDL-C/TC ratio in predicting the risks of CAD **(A)**, atherosclerosis **(B)**, and ischemic stroke **(C)**. We further evaluated the predictive performance of a PRS model combining the five lipid traits as well as that of another model combining all PRSs with sex and age. Abbreviations: PRS, polygenic risk score; AUC, area under the curve; TC, total cholesterol; LDL-C, low-density lipoprotein cholesterol; TG, triglyceride; HDL-C, high-density lipoprotein cholesterol; CAD, coronary artery disease; ROC curve, receiver operating characteristic curve; CI, confidence interval.

To evaluate the clinical applicability of PRSs, we used the PRS models developed using data from the CMUH cohort to predict various lipid-related diseases, such as coronary artery disease (CAD), atherosclerosis, and ischemic stroke (Figure 6). For CAD prediction, the AUC values of the PRS models based on TC levels, LDL-C levels, TG levels, HDL-C levels, and the LDL-C/TC ratio were 0.542, 0.540, 0.528, 0.451, and 0.529, respectively. When the PRS was combined with age and sex, the AUC value increased to 0.910 (Figure 6A). For atherosclerosis prediction, the AUC values of the PRS models based on TC levels, LDL-C levels, TG levels, HDL-C levels, and the LDL-C/TC ratio were 0.538, 0.538, 0.525, 0.455, and 0.527, respectively. When the PRS was combined with age and sex, the AUC value increased to 0.926 (Figure 6B). For ischemic stroke prediction, the AUC values of the PRS models based on TC levels, LDL-C levels, TG levels, HDL-C levels, and the LDL-C/TC ratio were 0.516, 0.517, 0.521, 0.472, and 0.514, respectively. When the PRS was combined with age and sex, the AUC value increased to 0.854 (Figure 6C). To quantify the contribution of the PRS models beyond that of traditional nongenetic risk factors, we evaluated the change in AUC (ΔAUC) by comparing a clinical baseline model (age and sex alone) with a combined model incorporating the PRS, age, and sex. As presented in Supplementary Table S13, the addition of the PRS to the baseline model significantly improved discriminative performance for all evaluated cardiovascular outcomes; the corresponding ΔAUC value was 0.04 for CAD, 0.04 for atherosclerosis, and 0.02 for ischemic stroke (all P_DeLong_ < 0.001).

To examine the clinical utility of genetic risk stratification, we analyzed disease prevalence across PRS deciles by using the lowest decile (bottom 10%) as the reference group (Supplementary Figure S4). Participants in the highest PRS decile (top 10%) exhibited significantly higher risks; the corresponding odds ratio (ORs) was 2.27 (95% confidence interval [CI]: 2.123–2.419) for CAD, 2.11 (95% CI: 1.979–2.252) for atherosclerosis, and 1.55 (95% CI: 1.444–1.664) for ischemic stroke.

## 4 Discussion

We conducted GWASs to identify genetic variants associated with TC levels, LDL-C levels, TG levels, HDL-C levels, and the LDL-C/TC ratio. Our analysis identified novel genes; although some of these are not listed in the GWAS catalog for the corresponding traits, they have previously been observed to be associated with lipid metabolism or other traits. For example, *EDEM3*, which was associated with TC levels in our analysis, has also been associated with TG levels (Xu et al., 2020). *ATXN1L*, which was associated with TG levels in our study, has also been associated with TC, LDL-C, and HDL-C levels (Sinnott-Armstrong et al., 2021; Richardson et al., 2020). In our analysis, four genes, namely, *APOC1*, *APOE*, *NECTIN2*, and *TOMM40*, exhibited significant associations with all the lipid traits. These findings are consistent with those of previous studies indicating that these genes are involved in lipid metabolism and CVD (Rouland et al., 2022; Yang et al., 2023; van der Graaf et al., 2020; Salakhov et al., 2014). *APOC1*, which inhibits lipoprotein lipase activity, affects cholesterol and TG levels and is associated with atherosclerosis risk (La Chica Lhoëst et al., 2024). *APOE* is essential for cholesterol transport and clearance, and its ε4 allele is associated with increased LDL-C levels and increased risks of Alzheimer’s disease and CVD (Deng et al., 2024). *NECTIN2*, a cell adhesion molecule, may indirectly affect lipid profiles by modulating liver cell function and inflammation-related lipid metabolism, and its genetic variants may contribute to atherosclerosis development (Li et al., 2021). *TOMM40*, which is located near *APOE*, modulates mitochondrial function and may affect lipid metabolism through fatty acid oxidation. Because of its role in mitochondrial processes, *TOMM40* has also been associated with Alzheimer’s disease risk (Yang et al., 2024; Hu et al., 2024). Our study findings indicate significant associations of *APOC1*, *APOE*, *NECTIN2*, and *TOMM40* with lipid traits in the Taiwanese Han population and demonstrate the genes’ relevance to lipid metabolism and related diseases.

In addition to the specific genetic loci identified, several methodological considerations related to our study design warrant discussion. The CMUH data were derived from a hospital-based cohort. Therefore, they are susceptible to selection bias and confounding from clinical variables because hospital patients typically have higher rates of metabolic comorbidities and are more likely to receive lipid-lowering treatment. Although adjustment for these factors and lifestyle variables as covariates would have been ideal, complete lifetime medication histories, comprehensive comorbidity profiles, and standardized lifestyle data were unavailable because of the decentralized nature of medical records across different institutions. To address these systemic data limitations and reduce pharmacological confounding, we intentionally used representative extreme measurements across multiple clinical visits (i.e., maximum values for TC, LDL-C, and TGs and minimum values for HDL-C). We selected these peak and nadir values because they may better reflect an individual’s pretreatment lipid profile and underlying genetic predisposition. Previous EHR-based lipid GWAS studies adopted similar approaches, such as the large-scale genomic investigation of blood lipids within the Million Veteran Program. To minimize potential confounding arising from the use of lipid-altering agents with variable patient adherence, the authors likewise utilized a strategy that used extreme lipid measurements (Klarin et al., 2018). The validity of this approach was strongly supported by our sensitivity analysis (Supplementary Figure S2), which demonstrated significant correlations between PRSs derived from extreme values and the participants’ mean clinical lipid levels. The robustness and generalizability of our model were further evaluated through independent external validation by using the community-based TWB cohort. Despite differences between the hospital-derived CMUH dataset and the community-based TWB population, genetic weights derived from the CMUH data maintained acceptable predictive accuracy across all the four lipid traits in the TWB cohort (Supplementary Figure S3). We note that the predictive performance in the TWB validation cohort was expectedly lower than that observed in our derivation cohort. This slight attenuation in performance can be primarily attributed to the inherent demographic and health-status differences between CMUH and TWB. Therefore, although residual confounding from unmeasured factors cannot be completely excluded, our analytical framework successfully reduced major therapeutic noise. The Taiwan-specific PRS demonstrated satisfactory predictive performance in both a hospital-derived patient population and a large-scale community-based population. These findings indicate the potential applicability of our Taiwan-specific PRS, suggesting that it may serve as a useful complementary tool for both clinical risk assessment in healthcare systems and proactive genetic screening for population-level health management.

Enrollment of large cohorts may improve the predictive accuracy of genetic assessments for disease risk; however, combining samples from different ancestral populations can introduce additional genomic complexities that can reduce model performance. One major factor underlying this limitation is the substantial variation in genetic architecture across populations, including differences in effect allele frequencies and LD patterns. In our study, despite accounting for between-population differences in alternative alleles, combining summary statistics from the global GLGC and CMUH datasets did not improve continuous correlation strength or AUC values, and the incorporation of only the external EAS cohort did not result in significant improvement (Figure 5). This lack of cross-ancestry transferability is supported by the marked directional discordance and statistical heterogeneity observed in our cross-cohort analysis (Supplementary Table S12). We identified thousands of genome-wide significant loci that exhibited completely reversed effect directions between the Taiwanese population and global cohorts, with a high proportion demonstrating substantial heterogeneity (I^2^ > 50%), which peaked at 38.7% for HDL-C levels and 25.6% for TC levels. A prominent example is the functional missense variant in *ABCG5* (rs6756629) associated with TC, for which the effect direction in our cohort was completely reversed relative to all reference populations. When weights from such highly discordant loci are combined in a multiancestry meta-analysis, the true localized genetic signals in the Taiwanese population can be partially obscured or diluted by the divergent LD patterns of non-local ancestries. These potential molecular-level discrepancies suggest that while global or broader regional East Asian weights offer substantial genomic insights, they may not fully capture the specific genetic characteristics of blood lipid traits in our localized population. Consequently, our findings support the incremental advantage and potential relevance of utilizing a population-specific PRS framework to optimize local risk assessment, rather than relying solely on global multiancestry composites. In terms of methodology, alternative algorithms specifically designed for cross-ancestry PRS construction, such as PRS-CSx and BridgePRS, have emerged to optimize risk prediction by integrating summary statistics from multiple diverse populations simultaneously. Rather than employing these simultaneous multi-ancestry tools, this study performed a comprehensive GWAS meta-analysis at the upstream discovery stage to capture shared trans-ancestry genetic effects. Subsequently, the standard PRS-CS method was applied to utilize these meta-integrated variant weights for localized LD refinement. This approach was specifically designed to maximize the statistical power of shared trans-ancestry genetic signals while preventing localized genetic structures from being diluted by extraneous ancestral LD patterns.

Lipid traits are associated with various metabolic diseases, such as hyperlipidemia, type 2 diabetes, and hypertension (van Dongen et al., 2013). This epidemiological pattern is supported by our PheWAS results, which reveal significant associations with various metabolic conditions. We were particularly interested in chronic kidney disease (CKD) and gout because these conditions are common in the Taiwanese Han population and may be associated with lipid traits. Our findings indicate that only the PRSs for TG and HDL-C levels were associated with CKD and gout. Notably, the PRS for HDL-C levels was negatively associated with these diseases (OR < 1), whereas the PRS for TG levels was positively associated with these diseases (OR > 1). In patients with CKD, dyslipidemia is characterized by decreased HDL-C levels and increased TG levels (Zhu et al., 2024). Although these PheWAS findings indicate shared genetic susceptibility between blood lipids and renal-metabolic disorders, the cross-sectional and observational nature of our analytical framework prevented us from clarifying the potential causal directions. Therefore, additional functional and longitudinal studies are required to clarify these directions.

Finally, the translational value of our Taiwan-specific PRS is supported by its utility in cardiovascular risk stratification when used in conjunction with clinical risk factors, rather than functioning as an independent predictive tool. Although traditional risk factors such as age and sex account for a substantial proportion of model discriminatory performance at baseline, the PRS still provides a statistically significant but relatively modest incremental contribution. Due to the lack of long-term cross-institutional tracking data for clinical events and formalized risk thresholds in CMUH dataset, calibration curves and reclassification metrics, such as the Net Reclassification Improvement (NRI) and Integrated Discrimination Improvement (IDI), were not calculated in this study. However, evaluating model performance on the basis of overall AUC improvements alone often overlooks the clinical implications for individuals at the extremes of the polygenic risk distribution. Using a decile-based approach (Supplementary Figure S4), we demonstrated a clear stepwise dose-response relationship in which individuals in the highest genetic risk tier (top 10%) had more than twofold higher risks of both CAD and atherosclerosis than did those in the lowest tier. This finding is highly consistent with the recent European Society of Cardiology clinical consensus statement, which demonstrated that although the incremental predictive improvement achieved by PRS appears modest at the population level, the primary clinical utility of PRS lies in enabling integrated risk stratification among individuals at the extremes of the PRS distribution (Schunkert et al., 2025). Therefore, we acknowledge that the overall clinical utility of the PRS as a standalone screening tool remains limited. However, when integrated alongside conventional risk factors like age and sex, our model can effectively help identify high-risk individuals within the Taiwanese population who would otherwise be misclassified as low risk, thereby enabling timely and personalized prevention.

## 5 Conclusion

This study identified key genetic variants associated with lipid metabolism in the Taiwanese population and demonstrated the importance of population-specific genetic insights. Although our PRS models exhibited satisfactory predictive performance for clinical risk stratification when used in conjunction with conventional risk factors, they should not be interpreted as independent diagnostic instruments. These findings should be cautiously interpreted within the limitations of hospital-derived data and cross-ancestry genetic heterogeneity. We also identified associations between lipid-related genetic factors and CVD risk, which may support the development of tailored screening and personalized risk management strategies. However, future prospective and functional studies are required to validate the clinical utility of these genetic risk assessments and clarify their underlying causal mechanisms.

## Data Availability

The datasets presented in this study can be found in online repositories. The names of the repository/repositories and accession number(s) can be found below: LocusZoom (Accession ID: 356962, https://my.locuszoom.org/gwas/356962/?token=5dcb199e1dec47cca27b1faaed28cdf4; Accession ID: 90495, https://my.locuszoom.org/gwas/90495/?token=8f9885ab1c104f85aaca51be3926eb06; Accession ID: 252569, https://my.locuszoom.org/gwas/252569/?token=0b48b8700a23405fb2f7352d0d01f383; Accession ID: 253555, https://my.locuszoom.org/gwas/253555/?token=bbd56effe97d4c80bada2e8fdb1fb390; Accession ID: 788288, https://my.locuszoom.org/gwas/788288/?token=b9a894cc9ae548dda4998fe25c600bb1; Accession ID: 937596, https://my.locuszoom.org/gwas/937596/?token=7858b0a177244db3a06d3463767862aa).
