# Supplementary material for: Evaluation of population-specific polygenic risk scores for blood lipids: insights from Taiwanese cohorts and multiancestry meta-analysis
Source: Front Bioinform. 2026 Aug 24;6:1823599. doi: 10.3389/fbinf.2026.1823599 (PMC13547480; doi:10.3389/fbinf.2026.1823599)
Supplement: Supplementary file 1 [file DataSheet1.docx]

Supplementary Figure 1. Flowchart depicting the GWAS pipeline.

The study cohort was divided into base and replication groups for PRS analysis. GWASs were conducted using data from the base group; the results were used to train the PRS models. A trans-ancestry meta-analysis was performed using GWAS results from five genetic ancestry cohorts. Abbreviations: PRS, polygenic risk score; GWAS, genome-wide association study; TC, total cholesterol; LDL-C, low-density lipoprotein cholesterol; TG, triglyceride; HDL-C, high-density lipoprotein cholesterol; AFR, Admixed African or African; EAS, East Asian; EUR, European; HIS, Hispanic; SAS, South Asia

Supplementary Figure 2. Scatter Plots of Correlations Between PRS-Predicted and Clinical Mean Lipid Levels.

Scatter plots illustrating the correlations between the model-predicted lipid levels (X-axis) and the actual clinically measured mean lipid levels (Y-axis), indicating the models’ performance in predicting blood lipid levels. The dashed line represents the linear regression line. To evaluate prediction error, the metric Diff was defined as [abs (actual − predicted) / actual]. A threshold of Diff > 0.1 suggests that the absolute difference between the predicted and measured levels exceeded 10%. The orange dots represent instances where Diff was > 0.1, whereas the blue dots represent instances where Diff was ≤ 0.1. Abbreviations: HDL-C, high-density lipoprotein cholesterol; LDL-C, low-density lipoprotein cholesterol; TC, total cholesterol; TG, triglyceride.

Supplementary Figure 3. Scatter Plots of Correlations Between PRS-Predicted and Measured Lipid Levels in the Independent External Validation Cohort (Taiwan Biobank).

Scatter plots illustrating the correlations between the model-predicted lipid levels (X-axis) and the actual measured lipid levels (Y-axis) within the independent external validation cohort (Taiwan Biobank), indicating the models’ performance in predicting blood lipid levels. The dashed line represents the linear regression line. To evaluate prediction error, the metric Diff was defined as [abs (actual − predicted) / actual]. A threshold of Diff > 0.1 suggests that the absolute difference between the predicted and measured levels exceeded 10%. The orange dots represent instances where Diff was > 0.1, whereas the blue dots represent instances where Diff was ≤ 0.1. Abbreviations: HDL-C, high-density lipoprotein cholesterol; LDL-C, low-density lipoprotein cholesterol; TC, total cholesterol; TG, triglyceride.

Supplementary Figure 4. Risk Stratification of Cardiovascular Outcomes Across Deciles of the Taiwan-Specific PRS.

Forest plots illustrating the odds ratios (ORs) and 95% confidence intervals (CIs) for developing cardiovascular diseases across the deciles of the Taiwan-specific PRS distribution. Risks were calculated using multivariable logistic regression adjusted for age and sex. The lowest decile (D1, bottom 10%) serves as the reference group (OR = 1.0, represented by the dotted vertical line). The plots display the risk stratification (from bottom to top: D2 to D10) for (A) coronary artery disease (CAD), (B) atherosclerosis, and (C) ischemic stroke. Participants in the highest PRS decile (D10, top 10%) exhibited significantly elevated risks for all three conditions compared to those in the lowest decile. Statistical significance is indicated as: * p < 0.05, ** p < 0.01, and *** p < 0.001. Abbreviations: CAD, coronary artery disease; D, decile.
